# Assessing the Potential Synergistic/Antagonistic Effects of Citrinin and Cannabidiol on SH-SY5Y, HepG2, HEK293 Cell Lines, and Human Lymphocytes

**DOI:** 10.3390/toxins16120534

**Published:** 2024-12-11

**Authors:** Dubravka Rašić, Antonio Zandona, Maja Katalinić, Martin Češi, Nevenka Kopjar

**Affiliations:** 1 Division of Toxicology, Institute for Medical Research and Occupational Health, HR-10 000 Zagreb, Croatia; azandona@imi.hr (A.Z.); mkatalinic@imi.hr (M.K.); nkopjar@imi.hr (N.K.); 2 Independent Researcher, Kauzlarićev Prilaz 9, HR-10 000 Zagreb, Croatia; martin.cesi@gmail.com

**Keywords:** *Cannabis sativa*, cell viability, contaminants, cytotoxicity, genotoxicity, hemp, moulds, mycotoxins

## Abstract

The increasing use of *Cannabis sativa* products for medicinal, dietary, and recreational purposes has raised concerns about mycotoxin contamination in cannabis and hemp. Mycotoxins persist in these products’ post-processing, posing health risks via multiple exposure routes. This study investigated cytotoxic and genotoxic interactions between cannabidiol (CBD) and the mycotoxin citrinin (CIT) using human cell models: SH-SY5Y, HepG2, HEK293, and peripheral blood lymphocytes. IC_50_ values and membrane disruption were initially assessed, followed by an evaluation of genotoxicity in lymphocytes using the Comet Assay and Cytokinesis Blocked Micronucleus Cytome Assay. Obtained findings demonstrate that cell-type sensitivity varied across treatments, with combined CBD and CIT exposure exhibiting distinct interactions. Lactate dehydrogenase (LDH) release remained minimal, suggesting cytotoxicity did not stem from membrane disruption but likely involved intracellular pathways. In lymphocytes, CBD alone produced negligible cyto/genotoxic effects and weak antiproliferative responses, whereas CIT displayed clear toxic impacts. DNA damage indicates that CIT may induce genome instability through indirect mechanisms rather than direct DNA interaction, with evidence of potential aneuploidic effects from the CBMN Cyt Assay. Combined exposure led to a reduction in CIT-induced DNA and cytogenetic damage, suggesting CIT’s potential interference with the beneficial properties of CBD. These results provide a foundation for further toxicological assessments and highlight the necessity of standardized mycotoxin monitoring in cannabis-derived products.

## 1. Introduction

Moulds and their toxic secondary metabolites mycotoxins can contaminate food and feed during growth, transport, or storage. These compounds have been long known for their widespread occurrence and harmful effects, including carcinogenic, immunotoxic, hepatotoxic, nephrotoxic, and mutagenic properties [1]. The widespread occurrence of moulds has recently drawn researchers’ attention to study their presence in new plants used in food and medicine, including medical cannabis. 

The *Cannabis sativa* plant, with two subspecies, is grown for different purposes. Industrial hemp (*C. sativa L.* subsp. *sativa*) is cultivated for fiber and seeds, containing <0.3% THC (by dry weight), while subspecies *C. sativa L.* subsp. *indica* (Indian hemp or, popularly called marijuana) grown for recreational purposes contains >0.3% of THC [2]. 

The *C. sativa* plant contains various natural compounds such as terpenes, flavonoids, fatty acids and more than 100 cannabinoids [3]. In recent years, the cultivation of hemp has grown due to its increased use in the production of functional food and cosmetic products, auxiliary medicinal agents for the relief of various diseases, especially those used to reduce the side effects of chemotherapy and for recreational purposes [4,5]. Warm and humid conditions favorable for mould growth and cultivation of *C. sativa* plant make mycotoxin contamination in hemp a growing concern [3,6]. With the increase in the use and production of hemp (and marijuana), the number of studies on residues such as pesticides or mycotoxins has also increased because the presence of mycotoxins can pose a significant risk to immunocompromised hemp users [2,4,7,8]. 

Cannabis and hemp can be consumed in different forms, including dried flowers that are smoked, inhaled, or vaped; flowers processed for cannabinoid extraction; and formulated concentrates such as capsules, gels, creams, suppositories, and tinctures [2]. Exposure to mycotoxins can occur through ingestion, skin contact, or inhalation in both humans and animals [1]. Many mycotoxins are chemically stable enough to withstand processing or extraction, which means they can persist in contaminated hemp flowers and remain present in hemp products or even become more potent due to increased concentration [9,10].

In a study by Punja et al. (2019), over 22 different fungal and oomycete species on cannabis plants grown indoors and outdoors were found [11]. In a study in which the microbiome of hemp and marijuana flowers was investigated, 25 different fungal genera and more than 40 fungal species were found, most of them with medium or high mycotoxin production potential [2]. The main fungi genera found on cannabis plants belong to the *Aspergillus* genera, known for producing a wide range of mycotoxins such as aflatoxins, ochratoxin A (OTA), sterigmatocystin, patulin, citrinin (CIT) and others [12]. Likewise, in most of the studies on mycotoxins in hemp, the presence of the most toxic mycotoxins, aflatoxins and OTA, but not specified for CIT, was reported [2,7]. Since OTA in moderate climate regions usually co-occurs with many different mycotoxins, it is likely that some other mycotoxins such as CIT which contaminates a range of plant-based products can be found in hemp samples [13]. Besides *Aspergillus* moulds, nephrotoxic mycotoxin CIT can be produced by several fungal species also from the *Penicillium* and *Monascus* genera [14,15]. Although CIT is less toxic and less studied than OTA or aflatoxins, it can interact with OTA in additive, synergistic, or antagonistic ways depending on the dose and model used [16,17,18].

There is a legitimate concern about the presence of contaminants, including mycotoxins, in cannabidiol oil (CBD) and other supplements, but studies on monitoring mycotoxins in these products are rather scarce; on the other hand, the human health risk assessment methods commonly used for regulating food and pharmaceuticals have not yet been standardized for the emerging cannabis and hemp industries [2]. Hemp is most often contaminated with aflatoxins and OTA, both of which are carcinogenic or potentially carcinogenic [1]. Analysis of mycotoxins in CBD-based supplements that are commercially available online showed the presence of 16 different, mostly unregulated, mycotoxins [8]. A sequencing survey of fungal communities on cannabis flowers was conducted due to concerns about foodborne illnesses. The survey identified several mycotoxigenic fungal species, including *Penicillium citrininum* and *P. paxilli* [10]. As CIT is a mycotoxin that interferes with Ca^2+^ efflux in the mitochondrial permeability transition pore, it could compete with the same pathway targeted by CBD. This overlap raises concerns about potential CBD-CIT interactions in patients treated with CBD therapeutics [10,16,19].

In the present study, using human neuroblastoma (SH-SY5Y), hepatocellular carcinoma (HepG2), human embryonic kidney (HEK293) cell lines, and human peripheral blood lymphocytes we assessed the potential synergistic/antagonistic effects of CIT and CBD. The experimental design included two parts: In the first part on human hepatocytes, neural and kidney cells, cytotoxicity of CIT and CBD were screened and IC_50_ values were determined, along with the membrane disruption phenomena. In the second part, using Comet Assay and Cytokinesis Blocked Micronucleus Cytome Assay on the Human Peripheral Lymphocyte model, we further studied the genotoxicity of single compounds and their combination. We believe that overall results would bring novel and useful evidence essential for risking the assessment of both compounds.

## 2. Results and Discussion

### 2.1. Cell Viability

**Figure 1:**
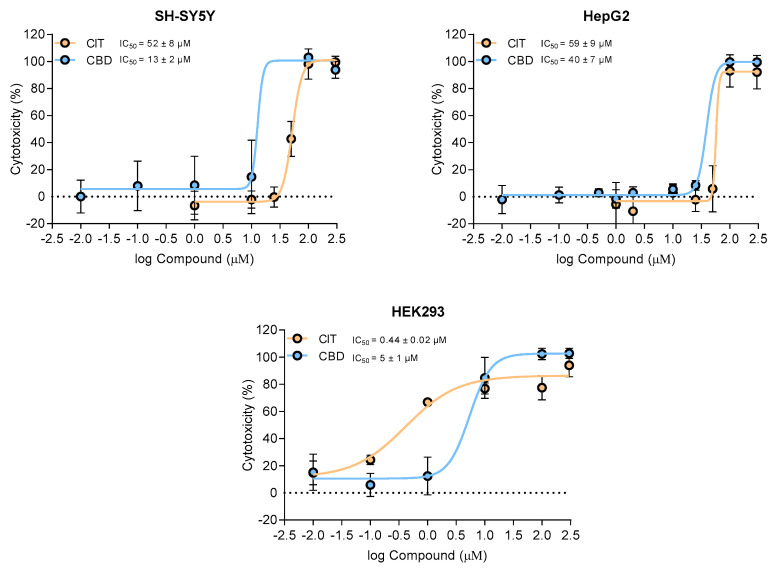
Dose-dependent cytotoxicity and IC_50_ values of cannabidiol (CBD) and citrinin (CIT) on SH-SY5Y, HepG2 and HEK293 cells after 24 h exposure. Experimental data are presented as a mean (±SE) of at least three experiments.

The possible cytotoxic effect of CBD and CIT was assessed in SH-SY5Y, HEK293, and HepG2 cell lines after a 24 h exposure period. The concentration range was selected with a focus on physiologically relevant concentrations and according to previous experiments on CIT [20,21]. The results obtained are presented in Figure 1, with corresponding IC_50_ values. Toxicity depended on compound and cell type, but all determined IC_50_ values were in the micromolar range. In HEK293 cells, CIT (IC_50_ = 0.44 ± 0.02 μM) displayed notably higher cytotoxicity than CBD (IC_50_ = 5 ± 1 μM), with IC_50_ values 11-fold lower than those for CBD, potentially due to receptor interactions or metabolic pathways more sensitive to CIT’s effects [3,22]. Contrastingly, HepG2 cells were most resistant to both compounds, possibly due to their robust detoxifying systems and xenobiotic metabolism, which may mitigate CIT’s (IC_50_ = 59 ± 9 μM) and CBD’s (IC_50_ = 40 ± 7 μM) cytotoxic effects, as observed in other hepatic models [23]. In SH-SY5Y cells, CBD (IC_50_ = 13 ± 2 μM ) was more cytotoxic than CIT (IC_50_ = 52 ± 8 μM), albeit both in the micromolar range, aligning with previous findings that highlight CBD’s pronounced effects in neuroblastoma and other neuronal cell models [24]. Interestingly, CBD’s cytotoxicity was 1.5- to 4-fold higher than CIT in both HepG2 and SH-SY5Y, reinforcing the compound’s varied cellular interaction profile [25]. 

**Figure 2:**
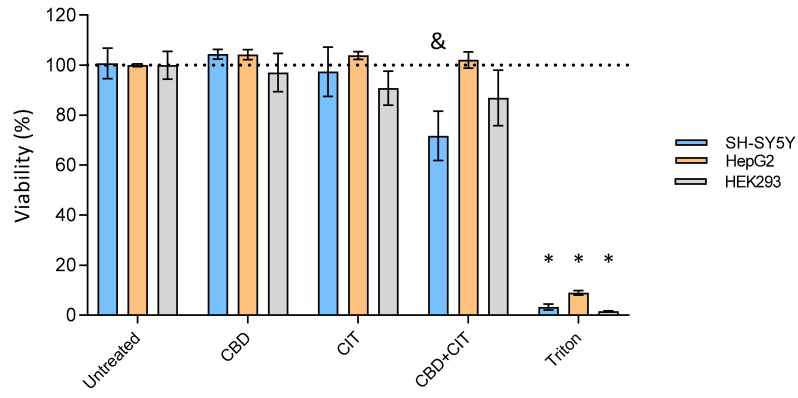
Viability of SH-SY5Y, HepG2 and HEK293 cells after 24 h exposure to cannabidiol (CBD), citrinin (CIT) and their combinations at lowest-observed-adverse-effect level (LOAEL) concentrations (1 and 30 μM for SH-SY5Y and HepG2 and 2 and 0.05 μM for HEK293, respectively). Experimental data are presented as a mean (±SE) of at least two experiments. The results are expressed as percentages of corresponding control, untreated cells, and given as means ± SE. & *p* < 0.05; * *p* < 0.0001 vs. untreated control.

Further on, to explore potential synergistic effects, cells were exposed to CBD and CIT at concentrations approximating the lowest observed adverse effect level (LOAEL). Consistent with the previous literature, CBD and CIT at their respective LOAEL doses did not independently impact cell viability (Figure 2), which aligns with findings that cannabinoids often exhibit non-toxic or minimal toxicity effects at lower concentrations [3,26]. However, the combination of CBD and CIT significantly reduced viability in SH-SY5Y cells, suggesting a potential synergistic interaction that may amplify cellular stress responses, particularly in neuroblastoma cells where cannabinoid effects on cell survival pathways, including oxidative stress and apoptosis, have been documented [27]. Although HEK293 cells displayed a similar viability reduction trend, the effect was not statistically significant, potentially due to variability in cellular response or a higher threshold for cannabinoid-induced toxicity in these kidney-derived cells [28]. For HepG2, lower cytotoxicity was not observed after exposure to CBD and CIT in combination. These findings emphasize the importance of combination treatments and indicate that interactions between cannabinoids may enhance their cytotoxicity in certain cell types, warranting further mechanistic studies to explore how cannabinoid combinations influence cell-specific pathways.

### 2.2. Cell Membrane Integrity

**Figure 3:**
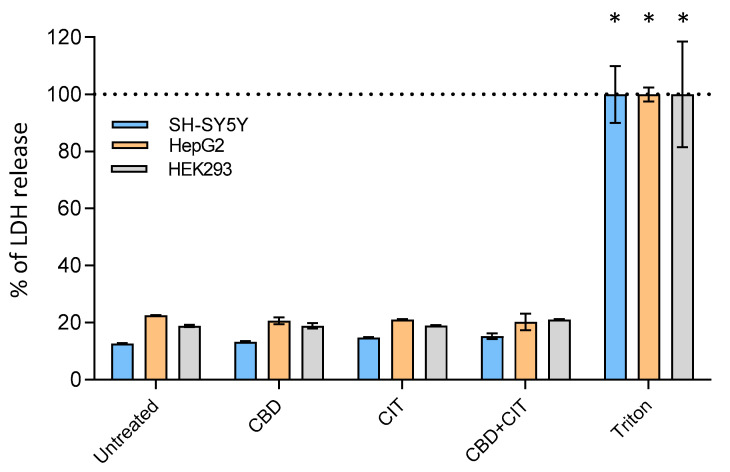
Levels of lactate dehydrogenase (LDH) release after 24 h exposure of SH-SY5Y, HepG2 and HEK293 cells after 24 h exposure to cannabidiol (CBD), citrinin (CIT) and their combinations at LOAEL concentrations (1 and 30 μM for SH-SY5Y and HepG2, and (2 and 0.05 μM for HEK293, respectively). Triton (0.08%) was used as the positive control. Experimental data are presented as a mean of percentage of LDH release (±SE) of at least two experiments. * *p* < 0.0001 vs. untreated control.

Cell membrane integrity, as evaluated by lactate dehydrogenase (LDH) release, was not significantly compromised by CIT, CBD, or their combination within the tested concentrations range (Figure 3). The absence of higher LDH release suggests that membrane integrity remained largely unaffected, indicating that the observed cytotoxic effects were not due to direct membrane disruption [29]. These findings align with prior studies showing that cannabinoids often exert cytotoxic effects through intracellular pathways, such as apoptosis or autophagy, rather than through membrane lysis or necrotic cell death [30,31]. The slight increase in LDH release observed is consistent with a low level of cytotoxicity, as indicated in previous assays, supporting the idea that CBD and CIT cytotoxicity may involve alternative pathways that do not directly compromise membrane integrity [29]. This observation underscores the selective nature of cannabinoid-induced cytotoxicity, which can vary based on cell type, exposure duration, and concentration, as cannabinoids may selectively trigger intracellular death mechanisms while maintaining overall cell membrane stability [32].

### 2.3. Peripheral Blood Lymphocytes

#### 2.3.1. Alkaline Comet Assay

**Figure 4:**
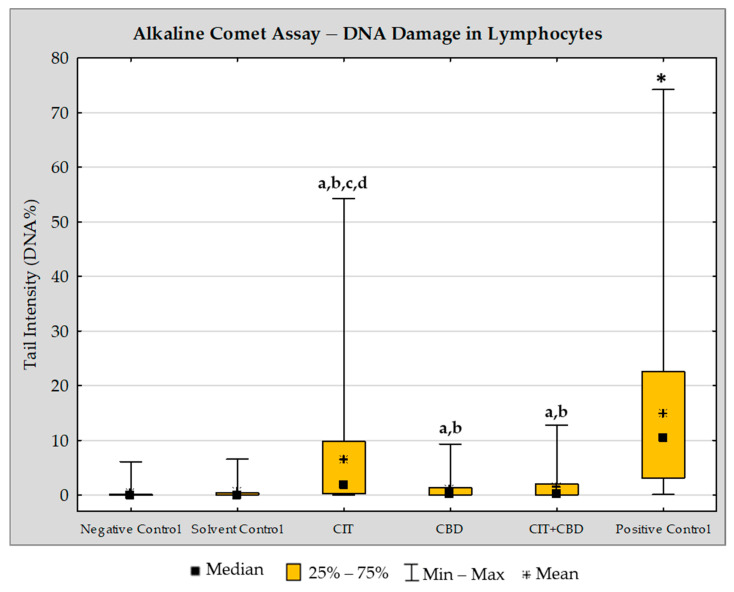
DNA damage in lymphocytes estimated by the alkaline comet assay. Lymphocyte cultures were treated for 24 h with citrinin (CIT) at 30 µM (7.50 µg/mL), cannabidiol (CBD) at 1 µM (3.15 µg/mL) and their combination (CIT + CBD, at the same concentrations). Negative controls (NCs) were non-treated lymphocytes. Solvent controls (SCs) were lymphocytes treated with ethanol (final concentration in the culture corresponded to 0.03%). Positive controls were lymphocytes treated for 24 h with bleomycin at 1.25 µg/mL. Six hundred independent comet measurements were carried out per experimental point. Results are expressed as mean/median, interquartile range, and range of measured values. Inter-group comparisons were performed using ANOVA with Tukey’s HSD post hoc test. Differences significant at *p* < 0.05 are marked with: *—vs. all other experimental groups; a—vs. negative control; b—vs. solvent control; c—vs. CBD; d—vs. CIT + CBD.

Results regarding the extent of DNA damage in lymphocytes estimated with the alkaline comet assay are shown in Figure 4. Exposure to single CIT resulted in DNA damage that differed from both controls with statistical significance at *p* < 0.05 (mean value ± SE of tail intensity was 6.56 ± 0.37%, compared to 0.52 ± 0.04% in the solvent control or 0.34 ± 0.04% in the negative control). Exposure to a single CBD slightly increased the value of tail intensity (1.07 ± 0.08%). Combined exposure to CBD and CIT resulted in a significant decrease in DNA damage compared to single CIT (1.38 ± 0.09%). The positive control sample had the highest mean value of tail intensity (14.81 ± 0.59%). Detailed inter-group comparisons and their statistical significance (ANOVA with Tukey’s HSD post hoc test, at *p* < 0.05) are marked in Figure 4. 

**Figure 5:**
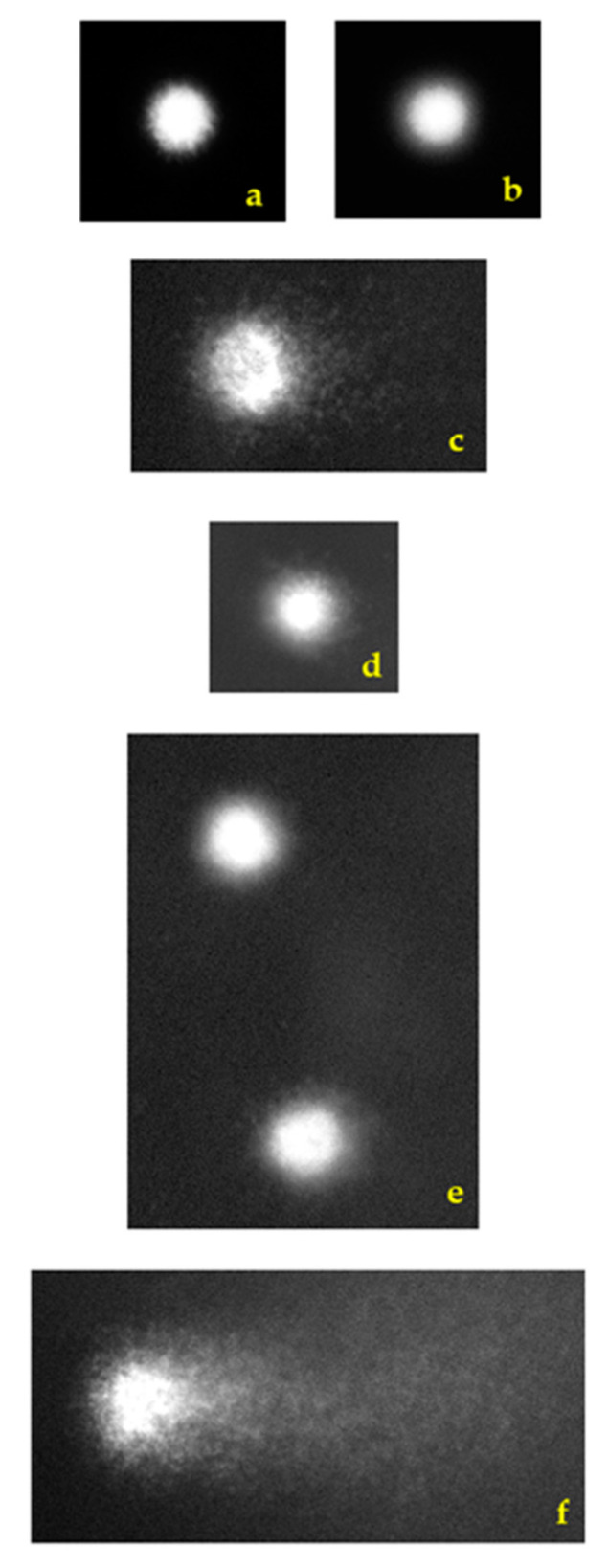
Typical lymphocyte nuclei observed under an epifluorescence microscope on microgels prepared for the alkaline comet assay. Non-damaged DNA in the negative control (**a**), and in the solvent control (**b**). Citrinin-treated lymphocyte (CIT) with damaged DNA (**c**). Low DNA damage in a cannabidiol-treated lymphocyte (CBD) (**d**), and in lymphocytes treated with combination of CIT and CBD (**e**). Highly fragmented DNA in a lymphocyte treated with bleomycin, positive control (**f**). Stained with ethidium bromide. Photomicrographs were taken under magnification ×200 using a black and white camera coupled with a computer-based image analysis system (Comet Assay IV, Instem-Perceptive Instruments Ltd., Suffolk, UK).

Typical features of lymphocyte nuclei observed using an epifluorescence microscope on microgels prepared for the alkaline comet assay are shown in Figure 5.

Here, we will briefly emphasize the significance of the findings obtained using the alkaline comet assay. This method allowed the assessment of DNA damage immediately after exposure to test compounds. Such damage may lead to cell death, may be repaired without persistent effect, may be fixed into a mutation, or may led to chromosomal damage [33]. So, understanding the immediate post-exposure DNA instability is important for predicting the extent of potential detrimental outcomes caused by test compounds, including their carcinogenic potential. When discussing the findings of the comet assay, it should be emphasized that most damage refers to DNA breaks originated from direct compounds interactions with DNA, from alkali labile sites (i.e., those without purinic/pyrimidinic bases), or appeared during excision repair processes (so called “transient” strand breaks) [34]. 

Obtained results indicate negligible genotoxic potential of CBD, and agree with previous observations obtained with comet assay in vitro [35,36,37,38,39]. In contrast, 24 h exposure to CIT led to measurable DNA damage in lymphocytes. This finding agreed with previous comet assay study by Stupin Polančec et al. [40]. The authors reported that 24 h exposure of HepG2 and A549 cells to CIT at its sub-IC_50_ and near IC_50_ concentrations induced significant DNA damage. Concentrations they tested were higher compared to ours. It has to be mentioned, indeed, the group median value of tail intensity we detected in CIT-treated sample was below 10%, pointing to a rather low overall level of DNA damage [41].

Some authors did not find significant DNA damaging effect of CIT using comet assay. Using specific modification of comet assay with formamidopyrimidine-DNA glycosylase (Fpg) enzyme, CIT at concentrations ≤60 μM for 24 h did not show toxic effect on HEK293 cells [42]. Additionally, no significant genotoxicity of CIT in HepG2 cells was observed using the comet assay [43]. It was reported that DNA damage caused by CIT in HepG2 cells was associated with oxidative stress rather than a direct interaction with DNA [44]. We agree with this observation. Obtained results of comet assay did not indicate statistically significant difference (at *p* < 0.05) between the values of tail intensity recorded in single CBD and CIT + CBD-treated cells. There is a possibility that the addition of CIT at the tested concentration was not able to significantly provoke DNA breakage. On the other hand, CBD, due to its known antioxidative properties, possibly counteracted detrimental effects of oxidative stress that led to indirect DNA damage caused by CIT. Such results imply that DNA damage caused by single CIT was not caused by direct interaction of CIT with DNA but rather by indirect mechanisms.

However, in the interpretation of results displayed in Figure 4, the emphasis on individual cells is important. As the other name of the method, i.e., “single cell gel electrophoresis” suggests, it is based on the assessment of damage in single, individual cells. As shown, the response of all treated cells to test compounds and their combination was not uniform. While single CIT was able to elicit a response in terms of different degrees of DNA damage in individual cells, after treatment with single CBD and CIT + CBD, the ranges of measured values were narrower. High tail intensity values in a certain proportion of CIT-treated cells mean that some cells with higher levels of DNA damage possibly may not be able to completely remove the resulting damage through their repair mechanisms, which could lead to mutations and increased cytogenetic risk. As the alkaline comet assay detects the extent of total primary DNA damage in individual cells, but does not offer information on cytogenetic effects and lymphocyte proliferation, we complemented the research with the Cytokinesis-Block Micronucleus Cytome (CBMN Cyt) Assay. This method is able to detect stable cytogenetic damage [45]. Using both approaches, we can determine the extent of repairable “primary” DNA damage and cytogenetic outcomes of non-repaired DNA damage induced by the test compounds.

#### 2.3.2. Cytokinesis-Block Micronucleus Cytome (CBMN Cyt) Assay 

**Figure 6:**
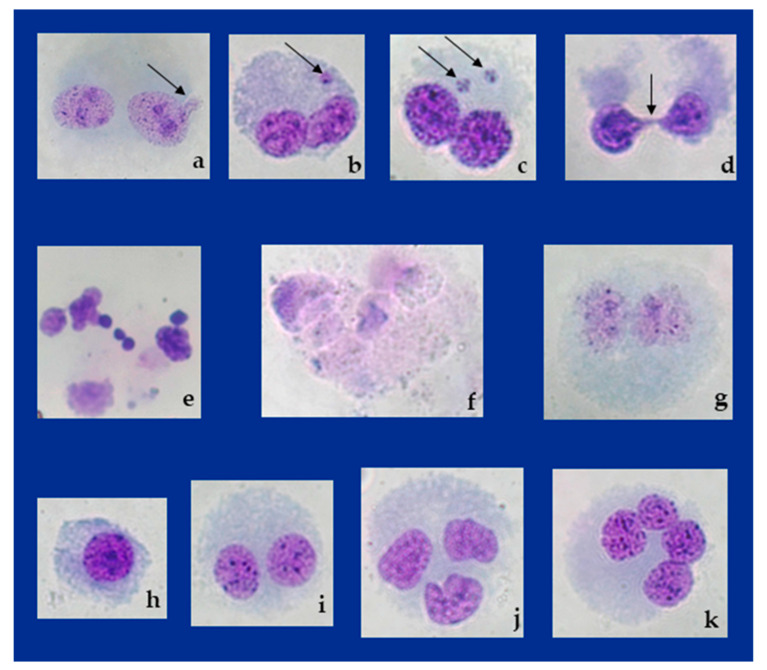
Photomicrographs of typical features observed on microscope slides prepared using Cytokinesis-Block Micronucleus (CBMN) Cytome Assay on human peripheral blood lymphocytes. Arrow indicates binucleated lymphocytes with (**a**) nuclear bud (NB) in the citrinin-treated sample (CIT); (**b**) micronucleus (MN) in the negative control sample; (**c**) two MNi in the citrinin + cannabidiol-treated sample (CIT + CBD); (**d**) nucleoplasmic bridge (NPB) in CIT-treated sample. Morphological features of dead cells in the positive control sample: (**e**) apoptotic cell with nuclear fragmentation; (**f**) a shift from apoptosis to necrosis; (**g**) necrotic cell. Typical features of cells scored to determine Cytokinesis-Block Proliferation Index: (**h**) mononucleated cell, M1 in negative control sample; (**i**) cell with two nuclei, M2 in negative control sample; (**j**) cell with three nuclei, M3 negative control sample; (**k**) cell with four nuclei, M4 in CIT-treated sample. Stained with Giemsa. Photographed at magnification ×1000 with Axiocam 208 color camera on Axiolab 5 microscope (Carl Zeiss Microscopy GmbH, Jena, Germany).

The results of CBMN Cyt Assay, along with detailed discussion of inter-group differences in terms of statistics are reported in Table 1. Negative and solvent controls had acceptable values compared to historical controls [46]. The highest values for all descriptors of the CBMN Cyt Assay were observed in the positive control. Citrinin at the tested concentration showed a high potential towards induction of MNi, NBs and NPBs compared to negative and solvent controls. Typical appearances of binucleated cells with these features observed on microscope slides are displayed in Figure 6.

Exposure to single CBD led to significantly lower levels of MNi, NBs, while no NPBs were observed. Combined exposure to CBD and CIT resulted in a significant decrease in all parameters studied with the CBMN Cyt Assay compared to single CIT. However, the addition of CIT induced formation of significantly more MNi compared to experimental group exposed to single CBD.

It is common knowledge that MNi originate from structural chromosome aberrations (especially fragments) or contain whole chromosomes that cannot rearrange into daughter cells. Process of nuclear budding occurs in the interphase, when surplus DNA amounts related to repair concentrated in the border nuclear region and expelled out. The NPBs predominantly originated from dicentric chromosomes that impair cell division in anaphase [47].

In view of our results, and previous reports by other authors, MNi produced by CIT treatment could be related to aneuploidogenic potential of the compound, rather to its direct effect on DNA/chromosomes. This is sustained by low primary DNA damage we measured by comet assay, and also by a low incidence of NPBs, that indicate structural chromosome aberrations. In their study, ref. [48] provided evidence on aneuploidogenic potential of CIT, and also specified that MNi in V79 cells treated with this mycotoxin contained whole chromosomes. Using the same cell model, cultured human lymphocytes, it has been shown that 48 h exposure to CIT at concentrations 60–100 μM, CIT resulted with significant induction of MNi in comparison with negative controls [49]. What made our research different from theirs was that we selected a shorter exposure time (24 h) and investigated the effects of exposure to a lower concentration of CIT (30 μM). Considering this fact, results of our research document that human lymphocytes already after 24 h of exposure showed a very high sensitivity to the harmful effects of CIT at a concentration twice as low as that one previously described in the same experimental model. 

Recent reports suggest that CBD does not produce significant biological response or genotoxic outcomes [36,50,51]. Štern et al. reported no changes in genomic instability, estimated by CBMN Cyt Assay after 24 h of exposure to CBD samples [36]. There was no significant increase in MNi, NBPs or NBUDs. Henderson et al. reported that CBD did not pose a genotoxic hazard in human TK6 cells up to 10–11 μg/mL, with and without metabolic activation [50]. Dziwenka et al. studied HempChoice^®^ product rich on CBD using human lymphocyte model and also did not find structural and/or numerical chromosomal damage [51]. Some authors, however, reported positive results of MN assay on human liver cell line (HepG2) and in buccal-derived cells (TR146), pointing to damage at levels above 0.2 µM and associated them with oxidative base damage [52]. We also assume, based on our own observations, that oxidative stress responses contribute to toxicity outcomes by both compounds tested here and intend to further investigate them in future studies.

In addition to the cytogenetic observed on lymphocyte model after treatment with CIT and CBD, we also have to briefly discuss the effects of those treatments on cell proliferation.

### 2.4. Analysis of Lymphocyte Proliferation 

**Figure 7:**
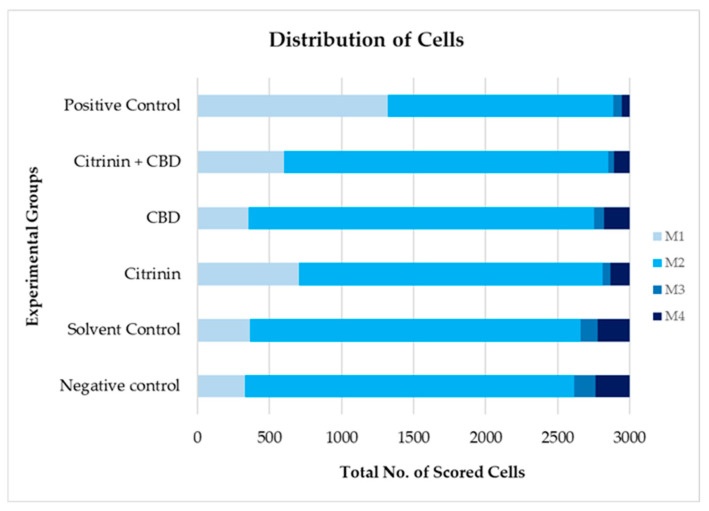
Results of analysis of lymphocyte proliferation in cell cultures treated in vitro for 24 h with citrinin (CIT) at 7.50 µg/mL (30 µM), cannabidiol (CBD) at 3.15 µg/mL (1 µM) and their combination (CIT + CBD, at the same concentrations). Negative controls were non-treated lymphocytes. Solvent controls were lymphocytes treated with ethanol (final concentration in the culture corresponded to 0.03%). Positive controls were lymphocytes treated for 24 h with bleomycin at 1.25 µg/mL.

Analysis of lymphocyte proliferation was based on scoring of cells with one nucleus (M1), two nuclei (M2), three nuclei (M3) and four nuclei (M4), whose typical features observed on the microscope slides are shown in Figure 4. Intergroup relations regarding the distribution of M1 to M4 cells are displayed in Figure 7.

To establish the number of cells with 1–4 nuclei (M1–M4), slides were analyzed using a light microscope at 400× magnification. Data are shown as the total number of cells scored in six independent evaluations (6 × 500 cells), per experimental group.

From Figure 7 it can be seen that lymphocyte proliferation was highly disturbed in the positive control, where the most of cells were mononucleated and binucleated. Since that sample was treated with cytotoxic drug bleomycin, such a result was expected. Inter-group comparisons revealed the statistical significance of the obtained results compared to all other samples (Pearson’s χ^2^ test, *p* < 0.05). 

More details regarding the statistical significance of results shown in Figure 5 can be seen in Table 2.

Treatment with single CIT, as well as combined treatment with CIT + CBD, resulted in specific patterns of cell distribution that also deviated from the negative and solvent control. In CIT-treated cultures, the proportion of M1 cells was significantly higher (Pearson’s χ^2^ test, *p* < 0.05) compared to the negative and solvent control, CBD-treated cultures, and compared to the cultures treated with CIT + CBD. In contrast, the proportions of M2, M3 and M4 cells significantly decreased compared to the negative and solvent control (Pearson’s χ^2^ test, *p* < 0.05). Furthermore, the proportions of M2 and M4 were significantly lower than those in the CBD-treated cultures, while the proportions of M2 were significantly lower compared to those in the cultures treated with CIT + CBD (Figure 7). 

Exposure to single CBD resulted in somewhat higher proportions of M2 cells compared to the negative and solvent control (Pearson’s χ^2^ test, *p* < 0.05), which produced consequent decreases in the proportion of M3 and M4 cells (Pearson’s χ^2^ test, *p* < 0.05). 

After the combined exposure, the range of scored cells was M2 > M1 > M4 > M3. The proportion of M1 cells was significantly higher (Pearson’s χ^2^ test, *p* < 0.05) compared to the negative/solvent control and CBD-treated cultures. In contrast, the proportions of M3 and M4 cells significantly decreased compared to the above-mentioned experimental groups.

The described fluctuations in M1 to M4 cell ratios affected the values of the Cytokinesis-Block Proliferation Index (CBPI) and Replication Index (RI), which were also used to estimate cytotoxicity in the present study. Individual values of both indices, calculated based on the obtained experimental data together with their statistical significances are shown in Table 3.

When we look at the results shown in Figure 7 and Table 3 together, the following can be concluded: treatment with single CIT caused a significant lowering of CBPI and RI compared to the negative/solvent controls, owing to increased proportions of M1 cells. Treatment with single CBD resulted in decreased CBPI and RI values compared to the negative/solvent controls, owing to increased proportions of M2, and decreased proportions of M3 and M4 cells. The values of CBPI and RI obtained for CIT + CBD-treated lymphocytes significantly deviated from single CBD, but not from single CIT. 

The value of RI of 83.46% determined after treatment with a single CIT means that, in terms of the cells that have divided to form bi- and multinucleated cells in the untreated culture, 83.46% of them were divided in the CIT-treated culture. As indicated by the value calculated for the combined exposure, RI = 85.74%, the replication of lymphocytes was not significantly affected by CBD and was predominantly disturbed by CIT. Therefore, the presence of both compounds caused detrimental effects on lymphocyte proliferation, which were confirmed by obtained values of CBPI, RI and cytotoxicity (%), reported in Table 3. 

As it stands, many publications report on the antiproliferative properties of CIT and CBD. Relevant facts and underlying mechanisms regarding the antiproliferative properties of CIT were presented in a comprehensive review, and documented on non-malignant cells, tumor cells, and animal models [53]. A recent study by Stupin Polančec et al. added more novel evidence regarding G2/M phase cell-cycle arrest in HepG2 and A549 cells following 24 h exposure to CIT at its sub-IC_50_ and near IC_50_ concentrations that were associated with the increase in total and phosphorylated Chk2 and FANCD2 checkpoint proteins [40]. There is growing evidence of antiproliferative effects of CBD as well [32,54,55,56]. 

Our study, which is rather descriptive, cannot provide definite answers to questions about specific mechanisms that could be considered responsible for the observed antiproliferative effects on lymphocytes, primarily due to limitations of the experimental model applied and the methods used. In general, obtained results indicate that potentially useful effects of CBD established on the lymphocyte model are modulated by the presence of CIT. This implies that CIT, when present as a contaminant of some CBD-rich product, could diminish the anticipated beneficial effects of CBD. Nevertheless, that could be further investigated on a much broader concentration range and using other experimental models.

The phenomena of cytotoxicity and cell death caused by CIT and CBD have been extensively investigated so far, and much is already known in that regard. Regarding apoptosis induction by CIT, findings of studies with other cell models suggest that it originates from stimulating cytochrome c release followed by activation of multiple caspases in HL-60 cells [57]. It was proposed that CIT induces apoptosis through ROS- and mitochondria-dependent pathways in mouse embryonic stem cells, together with the downregulation of survival signaling molecules, such as HSP90, Ras, Raf-1 and ERK-1/2 [58]. Chang et al. reported that CIT led to apoptosis via activation of ERK and JNK signaling pathways in the human embryonic kidney (HEK293) and HeLa cells [59]. Salah et al. (2017) reported that CIT induces apoptosis in human intestinal cell line HCT116 at least in part through induction of endoplasmic reticulum stress [60]. 

There are also several previous reports on CBD-induced apoptosis. For instance, using primary lymphocytes of mice, the apoptotic effect of CBD was closely associated with oxidative-stress-dependent activation of caspase-8 [61]. Using the hepatic stellate cell model, evidence regarding the pro-apoptotic effect of CBD mediated by the elevated basal level of endoplasmic reticulum stress was provided [62]. On breast cancer cells CBD treatment induces an interplay among PPARg, mTOR and cyclin D1 in favor of apoptosis induction [63].

Obviously, to elucidate the apoptotic potentials of CIT and CBD, different methods and approaches have been used so far, which differ from each other in terms of sensitivity and the possibilities of elucidating the mechanisms underlying cell death. In the present study, scoring of apoptotic and necrotic cells was performed simultaneously with scoring of other features of the CBMN Cyt Assay, on the same slides, based on the morphological features of the cells. Obtained results are reported in Table 1, while morphological features of dead cells are shown in Figure 4. Overall results indicate that apoptosis played a more significant role than necrosis in the removal of lymphocytes with DNA/cytogenetic damage induced by single and combined exposure to CIT and CBD. Exposure to a single CIT at the tested concentration significantly lowered lymphocyte viability, while single CBD treatment led to a lower rate of lymphocyte death. Combined exposure to CBD and CIT resulted in lower lymphocyte viability compared to the CBD-treated group but with no statistical significance. As anticipated, lymphocyte viability was most reduced in the positive control (Table 1).

Unfortunately, our experimental design cannot provide evidence regarding the origins of lymphocyte death, because it was based solely on the morphological discrimination of dead cells. Therefore, this has to be documented in future experiments we plan on the same cell model.

## 3. Conclusions

Using an in vitro approach, we studied the effects of CIT and CBD on several specific cell types. The range of tested concentrations and one exposure time allows us only a rough assessment of their effects and interactions, which will need to be further clarified through the application of different exposure scenarios, as well as the application of other more sensitive methods. Nevertheless, in this preliminary phase of the research, our main objective was to provide novel and interesting evidence that could represent a solid basis for planning future research and determine in which direction it must continue.

The exposure to CBD, CIT, and their combination varied by cell type, showing sensitivity under certain conditions, particularly in combination treatments. The lack of significant LDH release suggests that cytotoxic effects were not due to direct membrane disruption but likely involved intracellular pathways. These findings underscore the complexity of CBD-CIT interactions at physiological concentrations, highlighting the potential for selective cytotoxic effects based on cellular context and compound combinations.

Results obtained on the lymphocyte model suggest that exposure to a single CBD produced negligible cyto/genotoxic effects and weak antiproliferative effects in lymphocytes. Exposure to a single CIT under the same experimental conditions showed clear toxic effects using all methods. Findings of alkaline comet assay suggest that genome instability was mediated by indirect mechanisms of damage, rather than direct interaction of CIT with DNA. Most of the observed outcomes of CBMN Cyt Assay could be related aneuploidic effects of CIT. Combined exposure to both compounds resulted in a lowering of primary and cytogenetic DNA damage caused by CIT. Overall results of this pilot in vitro study suggest that CIT diminished the beneficial effects of CBD at the cell level. Therefore, the presence of CIT is not desirable and could be regarded as harmful. In that view, contamination of plants rich in CBD with CIT should be prevented and avoided. 

In view of all limitations, the open questions that remain should be a matter of forthcoming studies that should focus on other exposure scenarios and a much broader range of test concentrations, along with the application of sensitive biochemical methods able to document the impact of oxidative stress on the observed toxic effects. Furthermore, the cytogenetic part of the study has to be extended to methods that can prove the aneuploidogenic effects of CIT and relate them with specific chromosomes if possible.

## 4. Materials and Methods

### 4.1. Assessments on Cell Lines 

#### 4.1.1. Chemicals and Cells

##### CIT and CBD Solutions

Stock solutions of CIT purchased from Sigma-Aldrich (St. Louis, MO, USA) and CBD purchased from LGC Standards (Teddington, Middlesex, UK) were dissolved in 96% ethanol (Kemika, Zagreb, Croatia) in a concentration of 100 mM for cell treatment. Final concentrations from 0.05 to 30 μM were prepared in a growth medium without supplementation.

Human neuroblastoma SH-SY5Y cells (ECACC 94030304), human Caucasian hepatocyte carcinoma HepG2 (ECACC 85011430) and human embryonic kidney HEK293 (ECACC 85120602) used in the experiments were obtained from the European Collection of Authenticated Cell Cultures (ECACC) through Sigma-Aldrich (Steinheim, Germany). SH-SY5Y cells were grown in Dulbecco’s Modified Eagle’s Medium F12 containing 15% (*v*/*v*) fetal bovine serum (FBS), 2 mM glutamine, 1% (*v*/*v*) penicillin/streptomycin (PenStrep solution), and 1% (*v*/*v*) non-essential amino acids (NEAA solution). HEK293 and HepG2 cells were grown in EMEM medium (Sigma-Aldrich, Steinheim, Germany) supplemented with 10% (*v*/*v*) FBS, 2 mM glutamine, 1% (*v*/*v*) PenStrep and 1% (*v*/*v*) NEAA. All media and supplements were purchased from Sigma-Aldrich, Steinheim, Germany. Cells were cultured at 37 °C in a 5% CO_2_ atmosphere, the medium was changed every two days, and passage was carried out according to the manufacturer’s protocol.

#### 4.1.2. Viability Assay

MTS assay (CellTiter 96^®^ AQueous One Solution Cell Proliferation Assay, Promega, Madison, WI, USA) was used to determine the viability of cells/cytotoxic properties of tested compounds by measuring cells’ mitochondrial succinate dehydrogenase activity. The procedure followed a previously described protocol [64]. In brief, the cells were seeded in transparent 96-well plates at a density of 20,000 cells/well one day before the experiment and treated with tested compounds in concentrations: 0.01, 0.1, 0.5, 1, 2, 5, 10, 25, 50, 100 and 300 µM. Triton X-100 at a final concentration of 0.18% (*v*/*v*) (stock of 9% in water; Sigma-Aldrich, Steinheim, Germany) was used as a positive control. After 24 h, treatment with tested compounds and incubation with MTS reagent, the absorbance was read at 492 nm on an Infinite M200PRO (Tecan Group Ltd., Männedorf, Switzerland) plate reader. Data were taken from at least three independent experiments (each treatment performed in duplicate or triplicate) and expressed as percentages (mean ± standard error) of viable cells compared to control (untreated) cells. IC_50_ values were determined by a nonlinear fit equation predefined in Prism software 8 (GraphPad Software, San Diego, CA, USA). 

The same method was used to evaluate a potential toxic synergistic effect on cells exposed to a combination of CBD and CIT at approximately LOAEL doses (lowest observed adverse effect level, 1 and 30 μM for SH-SY5Y and HepG2, and 2 and 0.05 μM for HEK293, respectively). There is no official recommendation for CBD oral use, but according to the available literature, we used a dose that corresponded to daily intake, which was 10 mg/kg/day (calculated on a person of 70 kg ~ 1 μM) [20,21,65]. These selected LOAEL concentrations were used for all other assays as well.

#### 4.1.3. Cell Membrane Integrity

Cell membrane integrity was determined after exposure to compounds’ concentrations corresponding to LOAEL concentrations in 24 h by measuring the release of the intracellular lactate dehydrogenase (LDH) and the procedure followed a previously described protocol [66] using The CytoTox-ONE™ Homogeneous Membrane Integrity Assay (Promega, Madison, WI, USA). In short, the cells were seeded in black 96-well plates at a density of 10,000 cells/well one day before the experiment and treated with selected LOAEL concentrations. Triton X-100 at a final concentration of 0.18% (*v*/*v*) (stock of 9% in water) was used as a positive control to determine the maximal LDH release. Data were taken from at least two independent experiments (each treatment performed in duplicate or triplicate) and plotted as a percentage of LDH release compared to the determined maximal LDH release, according to the manufacturer’s calculation protocol.

### 4.2. Assessments on Human Peripheral Blood Lymphocytes

#### 4.2.1. Blood Sampling 

A peripheral blood sample was collected from a healthy male (age 32 years, non-smoker), free from exposure to genotoxic agents or medical irradiations for a year preceding blood collection. The donor was familiar with the details of the research and gave informed consent to voluntary participation. The collection of personal data and blood samples for the experiment was approved by the Ethics Committee of the Institute for Medical Research and Occupational Health, Zagreb, Croatia (Class: 01-18/23-02-2/1; Reg. No.: 100-21/23-13). A total of 40 mL of blood was drawn by venepuncture into lithium heparin (LH 170 I.U.)-coated tubes (BD vacutainer^®^, Becton Dickinson: BD-Plymouth, Roborough, Plymouth PL6 78P, UK). The whole amount of blood was used for the conduction of experiments immediately after collection.

#### 4.2.2. Experimental Design 

The experimental design included the establishment of lymphocyte cultures, and the 24 h treatments with the test compounds, performed according to the last OECD recommendations, given in the Test Guideline No. 487—In Vitro Mammalian Cell Micronucleus Test [67]. 

The selection of specific concentrations of test compounds adhered to the guidelines for in vitro genotoxicity testing using comet assay [68]. According to them, the selection of concentrations has to consider the cytotoxicity of the test compounds, in the way that the concentrations that decrease viability, compared to the concurrent control cultures, by more than 30% should be avoided. For that reason, in this pilot study, we evaluated the effects of CIT and CBD on lymphocytes at IC_20_ concentrations, established in the first part of the experiment on SH-SY5Y and HepG2 cell models. These concentrations were 7.50 µg/mL (i.e., 30 µM) for CIT and 3.15 µg/mL (i.e., 1 µM) for CBD. Solutions used in testing were prepared just prior to treatment by dissolving the test compounds in ethanol (Kemika, Zagreb, Croatia).

#### 4.2.3. Experimental Schedule

Two independent trials, with six experimental groups each, were conducted. A total of six replicate cultures for every single experimental group were established. This step was carried out as follows: aliquots of heparinized blood (V = 600 μL per culture) were pipetted into a growth medium RPMI-1640 (Gibco, Grand Island, NY, USA) supplemented with 10% fetal calf serum (Gibco, Grand Island, NY, USA) and antibiotics (penicillin and streptomycin; Sigma-Aldrich, Steinheim, Germany). Lymphocytes were stimulated with a mitogen phytohemagglutinin (Remel, Lenexa, KS, USA) in order to induce cell division prior to exposure to the test chemicals. Cultures were maintained in the humidified atmosphere of 5% CO_2_ at 37 °C (Heraeus Hera Cell 240 incubator; Langenselbold, Germany).

Negative controls (NCs) were non-treated lymphocytes. Solvent controls (SCs) were lymphocytes treated with ethanol (its final concentration in the culture corresponded to 0.03%). Positive controls (PCs) were lymphocytes treated for 24 h with bleomycin at 1.25 µg/mL (according to [69]). The other three groups were lymphocytes exposed to single CIT, single CBD, and their combination (CIT + CBD, at the same concentrations). 

Treatments with test compounds started 20 h after the initial establishment of the cell cultures and lasted for 24 h. Upon completion of the exposure, aliquots of 100 µL of the treated cultures were pipetted in sterile conditions and used to prepare microgels for the alkaline comet assay. The remaining content of flasks was further processed using Cytokinesis-Block Micronucleus Cytome (CBMN Cyt) Assay.

#### 4.2.4. Alkaline Comet Assay

Preparation of agarose microgels, lysis (at pH = 10), denaturation/electrophoresis (at pH > 13) and neutralization (at pH = 7.5) steps were performed in line with the basic alkaline comet assay protocol [70], and some minor adjustments proven earlier in our laboratory [71]. To measure the extent of DNA instability in single cells, slides were stained with ethidium bromide (20 µg/mL). They were analyzed using an epifluorescence microscope (Olympus BX51, Tokyo, Japan) under 200× magnification. A total of 600 randomly selected nucleoids (6 × 100) were measured per experimental point using Comet Assay IVTM software (version number: Comet Assay 4.2, TE4H-V245-UXIU-KF5N, Instem-Perceptive Instruments Ltd., Bury Saint Edmunds, UK). As the main descriptor of DNA damage, tail intensity (DNA% in the comet’s tail) was selected, as recommended by the current international compendium [72]. Data analysis, interpretation and reporting followed recommendations by [73].

#### 4.2.5. Cytokinesis-Block Micronucleus Cytome (CBMN Cyt) Assay

CBMN Cyt Assay was performed according to standard protocols proposed for micronucleus assay [45,67,74]. Upon completion of the exposure, lymphocyte cultures were subjected to centrifugation at 100× *g* using a benchtop centrifuge (ROTOFIX 32A, Hettich, Tuttlingen, Germany). During this process, cells settled to the bottom of the tube. The growth medium with tested compounds was removed by pipetting, discarded and replaced with a fresh amount of complete growth medium RPMI-1640 that contained Cytochalasin B (cytoB; Sigma-Aldrich, Steinheim, Germany) at a final concentration of 6 µg/mL. This procedure was timed to the 44^th^ hour of lymphocyte cultivation, as that time is a critical point for the addition of cytoB to inhibit cytokinesis and obtain binucleated cells. All treated and control cultures were further maintained in the previously described conditions at 37 °C until the end of the 72nd hour of cultivation, as proposed by protocols. Then, the preparation of microscopic slides started, which included harvesting of cells by centrifugation (100× *g*, ROTOFIX 32A centrifuge, Hettich, Tuttlingen, Germany), followed by hypotonic treatment (0.075 mol/L KCl; Kemika, Zagreb, Croatia), and a new round of centrifugation. The resulting sediments of cells were fixed in several repeating stages with methanol–acetic acid (3:1 *v*/*v*; both obtained from Kemika, Zagreb, Croatia) followed by centrifugation. The final lymphocyte suspension was dropped onto clean microscope slides. For their staining, a 5% aqueous solution of the Giemsa dye (Merck KGaA, Darmstadt, Germany) was used. Slides were analyzed at 1000× magnification using a light microscope (Leitz, Oberkochen, Germany). Morphological discrimination of MNi, NBs, NPBs, and cells in apoptosis or necrosis was performed in line with the recommendations of Fenech et al. [74] and Fenech [45]. To determine their frequencies, per experimental point 6 × 1000 BN cells were counted.

On the same slides, parameters of cell proliferation, cytostasis and cytotoxicity were studied, according to recommendations by OECD 2023 [67]. For that purpose, the numbers of mono-, bi-, tri-, and quadrinuclear cells (i.e., M1, M2, M3, M4) were scored at 400× magnification using a light microscope (Leitz, Oberkochen, Germany). The total number of M1 to M4 cells counted per experimental group was 3000 (i.e., 6 × 500 cells).

Based on the scored ratios of M1 to M4 cells, the Cytokinesis-Block Proliferation Index (CBPI) was calculated, using the formula: CBPI = [(No. of mononucleated cells) + (2 × No. of binucleated cells) + (3 × No. of multinucleated cells)]/(Total number of cells). 

To establish the relative number of cell cycles per cell during the period of exposure to cytoB in treated compared to control cultures, the Replication Index (RI) was also calculated according to the formula:

RI = {[(<No. binucleated cells> + <2 × No. multinucleated cells>)/(Total number of cells in treated culture)]/[(<No. binucleated cells> + <2 × No. multinucleated cells>) × (Total number of cells in control culture)]} × 100

Finally, from the obtained values of RI, the cytotoxicity estimate was deduced, as % Cytotoxicity = 100 RI.

Data analysis, interpretation and reporting followed recommendations by OECD 2023 [67].

#### 4.2.6. Statistics

Data were analyzed using the Statistica–Data Science Workbench software, version 14.0.0.15. (TIBCO Software Inc., Palo Alto, CA, USA). In the processing of alkaline comet assay data, basic descriptive statistical parameters (mean, standard deviation, standard error, median, minimum and maximum values) were first determined. Further analyses included logarithmical transformation to normalize the distribution of data and one-way analysis of variance (ANOVA) followed by post hoc Tukey’s Honestly Significant Difference (HSD) test for multiple comparisons between groups. Data obtained in CBMN Cyt Assay were processed by the same statistical tests and using Pearson’s Chi-Square (χ^2^) test to establish the significance of differences in results obtained for lymphocyte proliferation. The level of statistical significance was set at *p* < 0.05.

## Figures and Tables

**Table 1:** Results of Cytokinesis-Block Micronucleus (CBMN) Cytome Assay on human peripheral blood lymphocytes. Lymphocyte cultures were exposed to citrinin at 7.50 µg/mL (30 µM), CBD at 3.15 µg/mL (1 µM) and their combination (CIT + CBD, at the same concentrations) for 24 h. Negative controls were non-treated lymphocytes. Solvent controls were lymphocytes treated with ethanol (final concentration in the culture corresponded to 0.03%). Positive controls were lymphocytes treated for 24 h with bleomycin at 1.25 µg/mL.

	Experimental Groups			Negative Control	Solvent Control	Citrinin	CBD	Citrinin + CBD	Positive Control
Descriptors									
Micronuclei (MNi) and BN_MN_ Cells	Mean (MNi)_1000_ ± SD			1.7 ± 0.8	2.0 ± 0.0	12.3 ± 1.6 ^a,b,d,e^	2.8 ± 0.8	5.8 ± 1.2 ^a,b,d^	24.7 ± 2.5 ^a,b,c,d,e^
	Total (MNi)_6000_			10	12	74 ^a,b,d,e^	17	35 ^a,b,d^	148 ^a,b,c,d,e^
	Mean (BN_MN_)_1000_ ± SD			1.7 ± 0.8	2.0 ± 0.0	11.7 ± 1.4 ^a,b,d,e^	2.8 ± 0.8	5.5 ± 0.8 ^a,b,d^	20.3 ± 2.9 ^a,b,c,d,e^
	Total (BN_NB_)_6000_			10	12	71^a,b,d,e^	17	33 ^a,b,d^	122 ^a,b,c,d,e^
	Distribution of BN_MN_ Cells with	1 MN		10	12	66 ^a,b,d,e^	17	31 ^a,b^	99 ^a,b,c,d,e^
		2 MNi		0	0	5	0	2	20 ^a,b,c,d,e^
		3 MNi		0	0	0	0	0	3
Nuclear Buds (NBs) and BN_NB_ Cells	Mean (NBs)_1000_ ± SD			2.0 ± 0.6	1.8 ± 1.0	13.2 ± 3.7 ^a,b,d,e^	4.2 ± 0.4 ^a^	4.3 ± 1.5 ^a,b^	18.2 ± 6.5 ^a,b,c,d,e^
	Total (NBs)_6000_			12	11	79 ^a,b,d,e^	25 ^a^	26 ^a,b^	109 ^a,b,c,d,e^
	Mean (BN_NB_)_1000_ ± SD			2.0 ± 0.6	1.8 ± 1.0	12.3 ± 3.1 ^a,b,d,e^	4.2 ± 0.4 ^a^	4.3 ± 1.5 ^a,b^	16.2 ± 5.0 ^a,b,c,d,e^
	Total (BN_NB_)_6000_			12	11	74 ^a,b,d,e^	25 ^a^	26 ^a,b^	97 ^a,b,c,d,e^
	Distribution of BN_NB_ Cells with		1 NB	12	11	69 ^a,b,d,e^	25 ^a^	26 ^a,b^	85 ^a,b,c,d,e^
			2 NBs	0	0	5	0	0	12 ^a,b,c,d,e^
Nucleoplasmic Bridges (NPBs) and BN_NPB_ Cells	Mean (NPBs)_1000_ ± SD			0	0	0.7 ± 0.5	0	0	1.5 ± 0.8 ^a,b,c,d,e^
	Total (NPBs)_6000_			0	0	4	0	0	9 ^a,b,c,d,e^
	Mean (BN_NPB_)_1000_ ± SD			0	0	0.7 ± 0.5	0	0	1.5 ± 0.8 ^a,b,c,d,e^
	Total (BN_NPB_)_6000_			0	0	4	0	0	9 ^a,b,c,d,e^
Cell Death	Mean (Apoptosis)_1000_ ± SD			1.0 ± 0.0	1.5 ± 0.5	8.3 ± 1.6 ^a,b,d,e^	3.2 ± 0.8 ^a,b^	3.8 ± 0.8 ^a,b^	15.7 ± 5.6 ^a,b,c,d,e^
	Total (Apoptosis)_6000_			6	9	50 ^a,b,d,e^	19 ^a^	23 ^a,b^	94 ^a,b,c,d,e^
	Mean (Necrosis)_1000_ ± SD			0.8 ± 0.4	0.5 ± 0.5	7.5 ± 0.8 ^a,b,d,e^	1.5 ± 1.0	3.0 ± 0.6	11.8 ± 3.6 ^a,b,c,d,e^
	Total (Necrosis)_6000_			5	3	45 ^a,b,d,e^	9	18 ^a,b^	71 ^a,b,c,d,e^

Microscopic evaluation was performed using a light microscope at 1000× magnification. To establish the frequencies of micronuclei (MNi), nuclear buds (NBs), and nucleoplasmic bridges (NPBs) altogether 6000 binucleated (BN) cells (1000 per replicate) were scored. BN_MN_ refers to binucleated cells that contain micronuclei; BN_NB_ refers to binucleated cells that contain nuclear buds; BN_NPB_ refers to binucleated cells that contain nucleoplasmic bridges. Data are expressed as mean ± standard deviation (SD) of six independent evaluations, per experimental group, and total values. Statistical significance of the results was evaluated using analysis of variance (ANOVA) with post hoc Tukey’s Honestly Significant Difference (HSD) test, and Pearson’s Chi-Square (χ^2^) test. The level of statistical significance was set at *p* < 0.05. The letters indicate significant differences between experimental groups. a—the relevant group differs with statistical significance from the negative control; b—the relevant group differs with statistical significance from the solvent control; c—the relevant group differs with statistical significance from the citrinin group; d—the relevant group differs with statistical significance from the CBD group; e—the relevant group differs with statistical significance from the citrinin + CBD group.

**Table 2:** Statistical significances of inter-group differences regarding the number of cells with 1–4 nuclei (M1–M4) shown in Figure 5. Evaluations were made by Pearson’s Chi-Square (χ^2^) test.

	M1 Cells	Negative Control	Solvent Control	Citrinin	CBD	Citrinin + CBD	Positive Control
M2 Cells							
Negative control			n. s.	*p* < 0.0001	n. s.	*p* < 0.0001	*p* < 0.0001
Solvent Control		n. s.		*p* < 0.0001	n. s.	*p* < 0.0001	*p* < 0.0001
Citrinin		*p* < 0.0001	*p* < 0.0001		*p* < 0.0001	*p* = 0.001	*p* < 0.0001
CBD		n. s.	n. s.	*p* < 0.0001		*p* < 0.0001	*p* < 0.0001
Citrinin + CBD		*p* < 0.0001	n. s.	*p* < 0.0001	*p* < 0.0001		*p* < 0.0001
Positive Control		*p* < 0.0001	*p* < 0.0001	*p* < 0.0001	*p* < 0.0001	*p* < 0.0001	
	**M3 Cells**	**Negative Control**	**Solvent Control**	**Citrinin**	**CBD**	**Citrinin** **+** **CBD**	**Positive** **Control**
**M4 Cells**							
Negative control			*p* = 0.0439	*p* < 0.0001	*p* < 0.0001	*p* < 0.0001	*p* < 0.0001
Solvent Control		n. s.		*p* < 0.0001	*p* = 0.0003	*p* < 0.0001	*p* < 0.0001
Citrinin		*p* < 0.0001	*p* < 0.0001		n. s.	n. s.	n. s.
CBD		*p* = 0.0031	*p* = 0.0134	*p* = 0.0105		*p* = 0.0166	n. s.
Citrinin + CBD		*p* < 0.0001	*p* < 0.0001	n. s.	*p* < 0.0001		n. s.
Positive Control		*p* < 0.0001	*p* < 0.0001	*p* < 0.0001	*p* < 0.0001	*p* < 0.0001	

Negative controls were non-treated lymphocytes. Solvent controls were lymphocytes treated with ethanol (final concentration in the culture corresponded to 0.03%). The citrinin group were lymphocytes treated in vitro for 24 h with citrinin at 7.50 µg/mL (30 µM). The cannabidiol (CBD) group were lymphocytes treated in vitro with cannabidiol for 24 h at 3.15 µg/mL (1 µM). The citrinin + CBD group were lymphocytes treated in vitro with cannabidiol and CBD (at the same concentrations tested in individual treatments). Positive controls were lymphocytes treated for 24 h with bleomycin at 1.25 µg/mL. M1 refers to cells with one nucleus; M2 refers to cells with two nuclei; M3 refers to cells with three nuclei; M4 refers to cells with four nuclei. The table lists the exact values obtained for the level of statistical significance of the differences between the experimental groups. The abbreviation n. s. indicates that there was no statistically significant difference between the experimental groups in terms of the number of cells with one to four nuclei.

**Table 3:** Results regarding the parameters of cytotoxicity in the lymphocyte cultures treated in vitro for 24 h with citrinin at 7.50 µg/mL, CBD at 3.15 µg/mL and their combination. Negative controls were non-treated lymphocytes. Solvent controls were lymphocytes treated with ethanol (final concentration in the culture corresponded to 0.03%). Positive controls were lymphocytes treated for 24 h with bleomycin at 1.25 µg/mL.

	Experimental Groups	Negative Control	Solvent Control	Citrinin	CBD	Citrinin + CBD	Positive Control
Descriptors							
Cytokinesis-Block Proliferation Index		2.02	1.99	1.83^↓nc, sc, cbd, &^	1.96^↓nc, sc^	1.85^↓nc, sc, cbd^	1.60^↓nc, sc, cit, cbd,&^
Replication Index (%)		100.00	97.44	83.46	97.14	85.74	58.75
Cytotoxicity (%)		0	2.56	16.54	2.86	14.26	41.25

In line with the Organisation for Economic Co-operation and Development (OECD) recommendations (2023), the Cytokinesis-Block Proliferation Index (CBPI) and Replication Index (RI) were determined using 500 cells per culture. To calculate their values, altogether, 3000 cells with one to four nuclei were scored in six independent evaluations (6 × 500 cells), per experimental group, at 400× magnification. Statistical significance of the results obtained for CBPI was evaluated using Pearson’s Chi-Square (χ^2^) test. Symbol ↓ refers to decreases that were statistically significant. The abbreviations next to the CBPI values indicate from which groups the relevant experimental group differs with statistical significance: nc—vs. negative control; sc—vs. solvent control; cit—vs. sample treated with citrinin; cbd—vs. sample treated with CBD; &—vs. sample treated with citrinin + CBD. Obtained *p* values for inter-group differences in CBPI values were as follows: citrinin vs. negative control *p* < 0.0001; citrinin vs. solvent control *p* < 0.0001; citrinin vs. CBD *p* < 0.0001; citrinin vs. citrinin + CBD *p* = 0.0007; CBD vs. negative control *p* < 0.0001; CBD vs. solvent control *p* < 0.0001; citrinin + CBD vs. negative control *p* < 0.0001; citrinin + CBD vs. solvent control *p* < 0.0001; citrinin + CBD vs. CBD *p* < 0.0001; positive control vs. all other experimental groups *p* < 0.0001.

## Data Availability

The original contributions presented in this study are included in the article. Further inquiries can be directed to the corresponding author(s).
